# A Review of Plasma Synthesis Methods for Polymer Films and Nanoparticles under Atmospheric Pressure Conditions

**DOI:** 10.3390/polym13142267

**Published:** 2021-07-10

**Authors:** Hyo Jun Jang, Eun Young Jung, Travis Parsons, Heung-Sik Tae, Choon-Sang Park

**Affiliations:** 1 School of Electronic and Electrical Engineering, College of IT Engineering, Kyungpook National University, Daegu 41566, Korea; bs00201@knu.ac.kr (H.J.J.); eyjung@knu.ac.kr (E.Y.J.); 2 GBS (Global Business Services) IT, The Procter & Gamble Company, Cincinnati, OH 45202, USA; trav.parson@gmail.com; 3 School of Electronics Engineering, College of IT Engineering, Kyungpook National University, Daegu 41566, Korea; 4 Department of Electronics and Computer Engineering, College of Engineering, Kansas State University, Manhattan, KS 66506, USA

**Keywords:** atmospheric-pressure plasma, solution plasma, plasma polymerization, polymer films, room temperature growth, nanoparticles

## Abstract

In this paper, we present an overview of recent approaches in the gas/aerosol-through-plasma (GATP) and liquid plasma methods for synthesizing polymer films and nanoparticles (NPs) using an atmospheric-pressure plasma (APP) technique. We hope to aid students and researchers starting out in the polymerization field by compiling the most commonly utilized simple plasma synthesis methods, so that they can readily select a method that best suits their needs. Although APP methods are widely employed for polymer synthesis, and there are many related papers for specific applications, reviews that provide comprehensive coverage of the variations of APP methods for polymer synthesis are rarely reported. We introduce and compile over 50 recent papers on various APP polymerization methods that allow us to discuss the existing challenges and future direction of GATP and solution plasma methods under ambient air conditions for large-area and mass nanoparticle production.

## 1. Introduction

In previous years, many of the papers and studies on polymer synthesis using the atmospheric-pressure plasma (APP) method have focused on specific application processes. However, there are very few review papers that provide a comprehensive view of the polymer synthesis methods using APP, which makes it difficult for new researchers in the field who want to try APP polymer synthesis. Beginning in the 1790s, the report written by N. Bondt et al. on polymer synthesis using electrical discharge [1] is seen as one of the first documented studies on plasma polymerization. In the 19th century, arc synthesis of hydrocarbons was studied by chemists such as de Wilde et al. [2], Berthelot et al. [3,4], and P. and A. Thenard [5]. Studies on the synthesis of various organics using glow discharge were first published by German scientists in the 1960s [6,7,8,9]. Afterwards, the first applications using these plasma polymers were reported by Goodman [10], and subsequent studies on the property improvements of materials using plasma polymers were actively conducted, with a focus on the interaction between plasma and various substances [11,12,13,14,15,16]. Today, plasma synthesis is selected for various applications, such as layer deposition for electrical devices [17,18,19,20], antibio- or bio-material applications [21,22,23,24], and surface modification [25,26,27,28], among others.

Plasmas are well known as ionized quasi-neutral gases consisting of photons, neutral particles, metastable particles, ions, radicals, and electrons. Plasmas with the same density of positive-charged particles (ions) and negative-charged particles (electrons) behave macroscopically neutrally in free space [29,30]. These plasmas can be artificially generated by waves, lasers, combustion, flames, and even nuclear reactions, though the most common approach for plasma generation is electrical discharge from electric power sources in the laboratory [31,32]. The plasma generated by electric power is called ‘non-equilibrium plasma’ because the temperature (or kinetic energy) of heavy particles, including ions, is different from that of the electrons. Only the electrons receive energy from the electric field generated by applied electric power; thus, the electron temperature (several thousand K) is relatively higher than the heavy particles’ temperature (around room temperature). This fact is responsible for the definition of such plasma generated by electric power as ‘cold plasma’ or ‘non-thermal plasma’ [32]. In the case of polymer synthesis, the non-thermal plasma method (or non-thermal plasma polymerization) can avoid thermal damage to substrates or substances due to the relatively low temperatures of the heavy particles. In addition, the energetic electrons with high energy supplied from plasma can produce high concentrations of reactive species or free radicals from precursors [32,33,34]. This method not only has the degree of freedom for material selectivity without insolubility problems, but also reduces or eliminates the need for an oxidant or reductant [35,36,37,38]. Overall, plasma polymerization presents several advantages, such as simple installation, high reactivity, high throughput, fast processing, low cost, low temperature, and green synthesis [39,40,41,42].

The non-thermal plasmas are roughly classified into vacuum plasma and APP, depending on the plasma working pressure required—of which APP has substantial potential for process enlargement because it does not require a high-quality vacuum system, which significantly reduces the overall setup and operating costs. Such APP can be obtained under atmospheric pressure conditions, avoiding extreme handling conditions [43]. For these reasons, polymer synthesis methods using APP have attracted growing attention in recent years, owing to their high potential for polymer deposition and nanoparticle (NP) synthesis for various applications [17,18,19,20,21,22,23,24,25,26,27,28]. Accordingly, attempts to generate plasma under atmospheric pressure have been successful, and various structures of plasma devices have been proposed [44,45,46,47,48]. Many reports have described the conditions needed to generate plasma, and the properties of plasma-synthesized polymers have been thoroughly investigated [25,39,42,45,49,50]. These studies may lead to further active investigations into methods and applications of this new plasma equipment in the near future.

**Figure 1:**
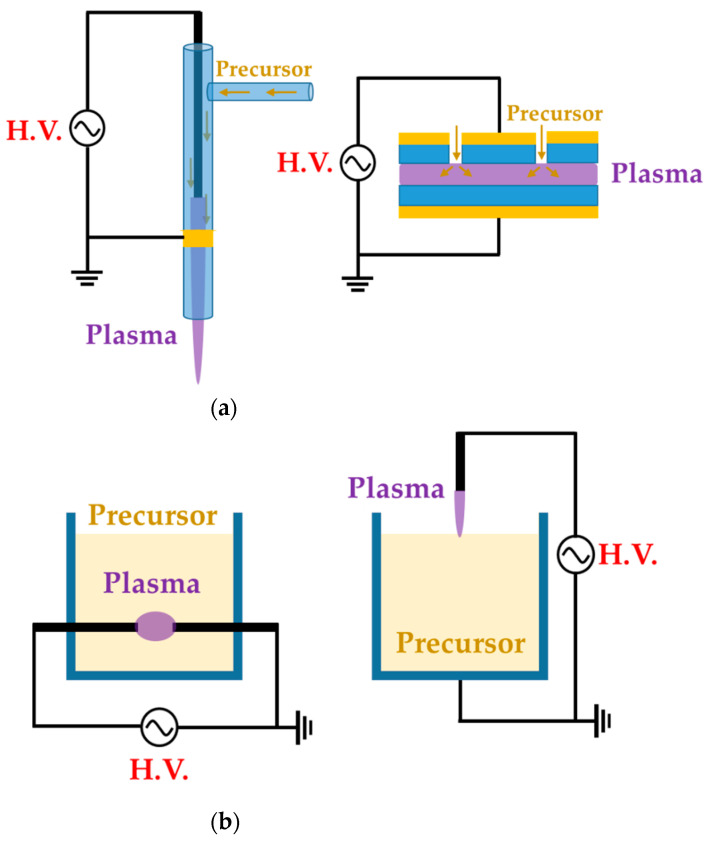
Representative configuration of (**a**) gas/aerosol-through-plasma (GATP) methods (left: jet type, right: dielectric-barrier discharge (DBD) type) and (**b**) solution plasma methods (left: in-solution plasma, right: on-solution plasma).

We divide these APP polymer synthesis techniques into two types, depending on the phase of employed precursors: the first is APP polymerization, using a gas- or aerosol-type precursor [21,39,41], while the second is when the solution itself is supplied as a precursor [38,43,44], as shown Figure 1. In this review, the former is referred to as the gas/aerosol-through-plasma (GATP) method, and the latter is denominated as the solution plasma method. For students and researchers starting out in the APP polymerization field, the aim of this review is to introduce an overview of recent studies on these polymer synthesis methods employed by various APP techniques for the formation of polymer films and NPs. Additionally, the methodological classification of APP polymer synthesis according to the precursor phase employed, using various kinds of plasma equipment, is included. The main purpose of this review paper is to provide a reference for recent APP devices for polymerization, while briefly discussing APP polymerization. We hope that students and researchers trying to synthesize plasma polymers can then select the best suited methods for their experiments.

## 2. Synthesis Method Using Gas/Aerosol-Type Precursors (GATP)

GATP methods use a discharge gas for generating the APP, and floated precursors that are in aerosol or gaseous form. Materials in a gaseous state at room temperature are themselves applied as precursors, whereas materials that exist as liquids are mainly introduced to a plasma region as an aerosol via atomizing or bubbling with gas. GATP methods are commonly used for the deposition of polymer films [51,52,53,54,55,56,57,58,59,60,61,62,63,64,65,66,67,68,69,70,71,72,73,74,75,76,77,78,79,80,81,82,83,84,85], as this method allows in-line processing by moving either the APP devices or the substrates [39].

### 2.1. Atmospheric-Pressure Plasma Jet (APPJ) Method

An atmospheric-pressure plasma jet (APPJ) is a device for polymerization that generates directional plasma from a narrow nozzle and a gas flow with high input energy. Polymerization with APPJ devices enables local processing, because the process area is limited to the jet plasma plume size [51,59]. A gas/aerosol precursor becomes activated (or fragmented) by passing through the plasma generation region, and then the fragments become neutral passive (or recombine) beyond the plasma stream end, and are deposited onto a substrate that lies outside the plasma region [86,87].

**Figure 2:**
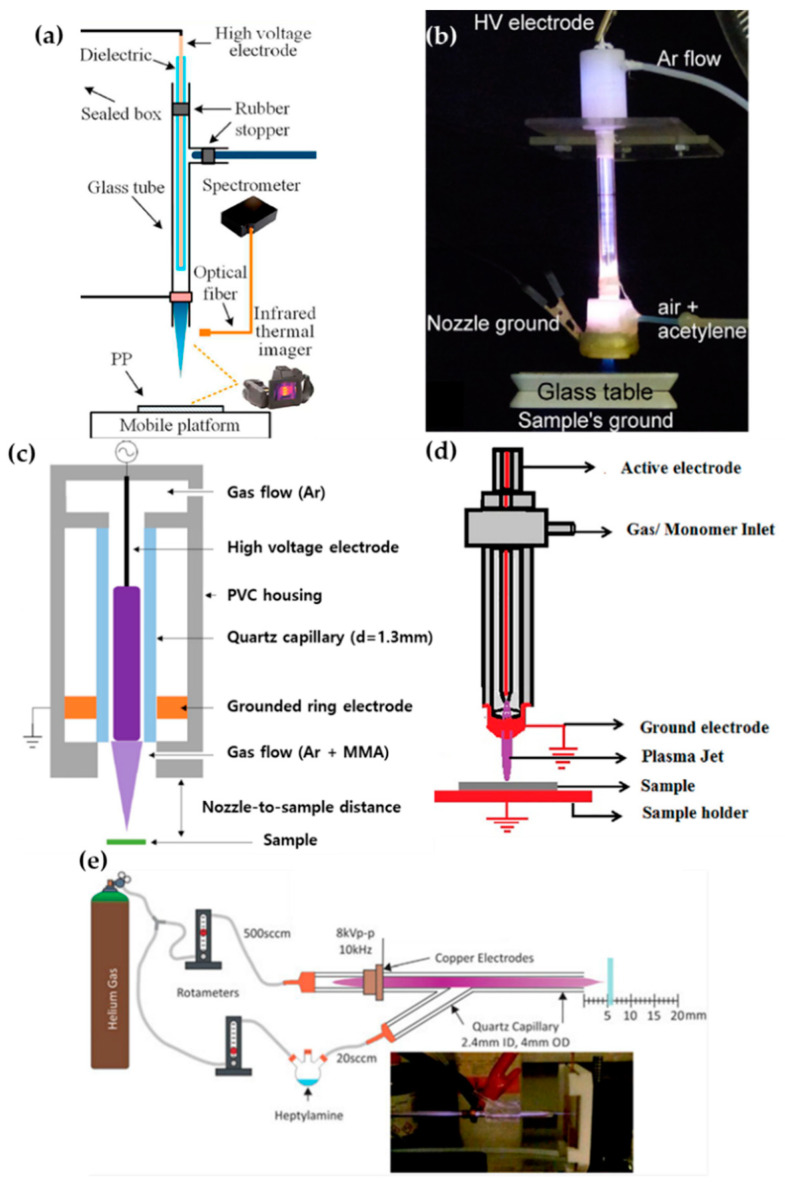
Structures of pin–ring-electrode-type APPJs from (**a**) Zhang et al. [51], (**b**) Ricci Castro et al. [52], (**c**) Van Vrekhem et al. [53], and (**d**) Pandiyaraj et al. [54], and (**e**) schematic of experiment using a y-shaped APPJ with a ring powered electrode, by Doherty et al. [55].

Zhang et al. [51], Ricci Castro et al. [52], Van Vrekhem et al. [53], and Pandiyaraj et al. [54] reported an APPJ with pin–ring electrodes. The pin electrode and the ring electrode are used as high-voltage (HV) and ground electrodes, respectively. Zhang et al. reported an APPJ for poly(methyl methacrylate) (PMMA) coating on a bumpy surface. This APPJ consists of a T-shaped quartz glass body and pin–ring electrodes; the pin electrode is a copper (Cu) rod covered with a quartz glass tube (Figure 2a). Both the plasma discharge gas and monomer bubbler gas are argon (Ar). In this method, the methyl methacrylate (MMA) monomer liquid is housed in a bottle within an oil bath held at 40 °C, and is bubbled with Ar gas introduced through the branch of the quartz body in an aerosol state. The power generation uses an alternating current (AC) source from 10 kHz to 60 kHz with a maximum voltage of 17 kV supplied through the pin electrode [51].

Ricci Castro et al. and Van Vrekhem et al. used a similar configuration of the APPJ for synthesizing plasma polymers: Two APPJs were utilized that both had tungsten pin electrodes; however, aluminum (Al) and copper (Cu) were used as grounded ring electrodes. As shown in Figure 2b,c, these APPJ devices consisted of three parts: the head of the device had an HV electrode and a discharge gas inlet; the body was constructed of a glass tube; and the final component was a plasma nozzle with a grounded ring electrode and precursor inlet. In the paper by Ricci Castro et al., Ar was supplied for discharging, and an air/acetylene mixture gas was supplied as a precursor. The power source was at 19 kHz frequency and 17 kVp–p (peak-to-peak) voltage, consisting of two sinusoidal waves with disparate amplitudes to avoid overheating issues [52]. In the paper by Van Vrekhem et al., Ar gas was also used as the discharge gas, while aerosol MMA was bubbled by the Ar gas and input into the plasma afterglow region; this utilized power generation from a 23-kHz AC HV source to activate the APP [53].

The APPJ of Pandiyaraj et al. was fed the discharge gas, with the monomer in the same path. Both the pin and ring electrodes were made of Cu; the pin Cu electrode was encapsulated by a quartz tube (Figure 2d). Triisopropyl phosphate (TIP) was vaporized by heating to a maximum temperature of up to 500 °C, and the Ar discharge gas carried the TIP vapor into the inlet. Here, the AC power (maximum voltage = 40 kV; current = 30 mA; and frequency = 50 kHz) was supplied to generate the APP [54].

Doherty et al. synthesized the plasma polymer of heptylamine onto polystyrene. They used a y-shaped quartz capillary as the APPJ body, with a single powered ring electrode. Helium gas (He) used for plasma discharge was introduced in the downstream flow, and heptylamine aerosol was bubbled by the He introduced into the stream via a branch off the quartz body (Figure 2e). The supplied power was a sinusoidal current with a voltage of 8 kVp–p and a frequency of 10 kHz [55].

**Figure 3:**
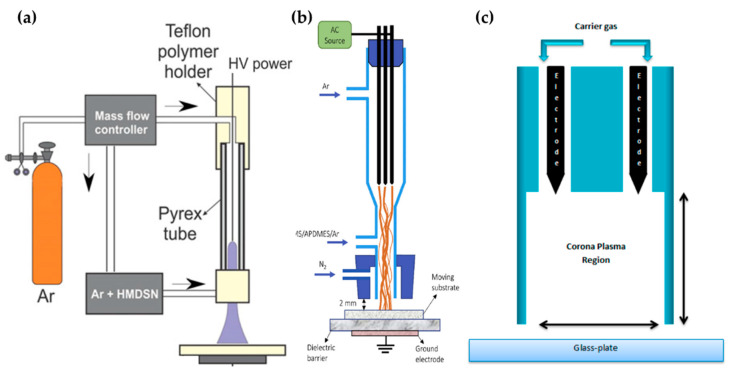
Structures of pin electrode type in the APPJs of (**a**) Kodaira et al. [56], (**b**) Hossain et al. [57], and (**c**) Malinowski et al. [58].

Kodaira et al. [56], Hossain et al. [57], and Malinowski et al. [58] proposed an APPJ with only pin-type electrodes for generating the APP, as shown in Figure 3. Kodaira et al. investigated the characterization of APP-polymerized hexamethyldisilazane (HMDSN); they used the same device as the studies from Ricci Castro et al. [52], but without a grounded ring electrode (Figure 3a). The power was also the same waveform, with 12 kVp–p. Both the discharge gas and HMDSN monomer carrier gas were Ar, which was introduced through the upper side and nozzle of the APPJ, respectively [56].

Hossain et al. reported the APP polymerization of tetramethylsilane (TMS) and 3-aminopropyl(diethoxy)methylsilane (APDMES) for superhydrophobic coatings on glass. The APP was generated from three pin electrodes, which were stainless steel needles arranged at 120° intervals in a DBD glass reactor. This glass had two branches as a gas inlet—the upper branch was the main Ar gas inlet for discharge, while the lower branch guided the precursor flow. A cap was attached to the nozzle component, and nitrogen gas (N_2_) was injected to shield the stream from interaction with the ambient air. A mixed liquid of TMS and APDMES was prepared as the precursor, and was bubbled with Ar (Figure 3b). This plasma reactor was served with AC power at a frequency of 11.5 kHz to generate the APP discharge [57].

A sinusoidal AC HV power source with a peak voltage of 2–6 kV and a frequency of 20 kHz was applied through two pin electrodes to generate He corona plasma for the deposition of laccase by Malinowski et al. (Figure 3c). The solution of laccase with 10% ethyl alcohol was then atomized by a nebulizer for precursor injection to the plasma region [58]. 

**Figure 4:**
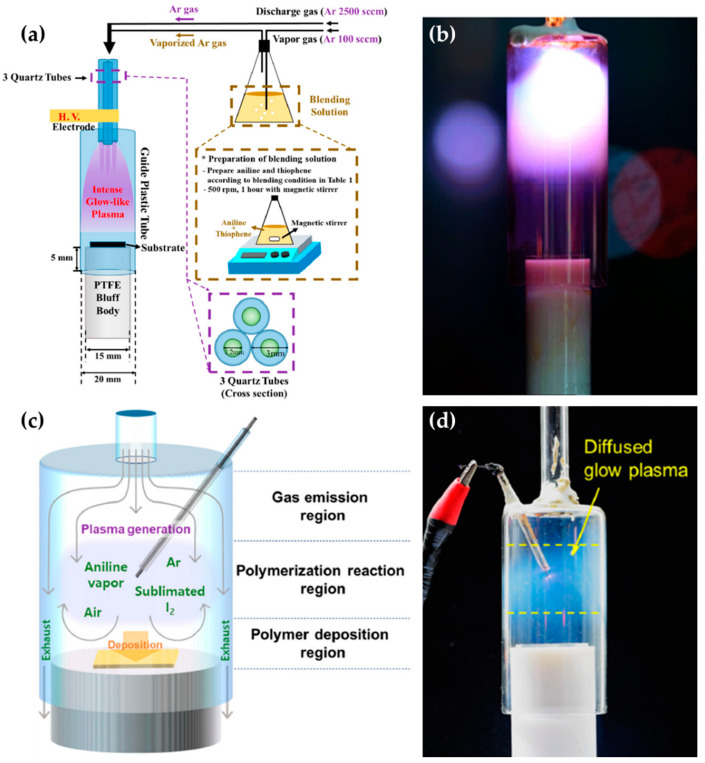
(**a**) Setup [59] and (**b**) photo image [62] of the APPJ with three array jets and a GB system. (**c**) Configuration [63] and (**d**) photo image [64] of the pin-type APPJ with a GB system.

The research groups of Jang et al., Park et al., and Kim et al. [59,60,61,62] proposed an APPJ with three array jets and a unique shielding system. This system is called the guide-tube and bluff-body (GB) system. The guide tube that blocks the plasma reactor from ambient air is attached to the tip of the array jets, and the bluff body serves to introduce a substrate into the guide tube. The three jets are wrapped with Cu tape as HV electrodes (Figure 4a). Thanks to the ambient air blocking and special internal flow of the GB system, it is possible to expand the area of high-density plasma by more than 60 times (Figure 4b) [62]. They synthesized the copolymer [59], pin-hole-free polymer [60], conducting polymer [61], and single-crystalline polymer [62] using this APPJ device.

Kim et al. report a pin-type APPJ with the GB system for synthesizing a conducting polymer [63] and a transparent polymer [64]. As shown in Figure 4c, this device consists of four components: a narrow glass for gas inlet, a wide glass tube as the guide tube, a polytetrafluoroethylene stand as the bluff body, and a tungsten wire electrode to generate plasma. The tungsten wire electrode is covered with a glass capillary, with just the 2-mm tip of the wire remaining exposed. A discharge gas and a precursor are introduced into the guide tube via the gas inlet. A sinusoidal power with a peak voltage of 4–5 kV and a frequency of 30 kHz is applied through the tungsten wire electrode, forming a diffused-glow plasma for polymerization (Figure 4d).

**Figure 5:**
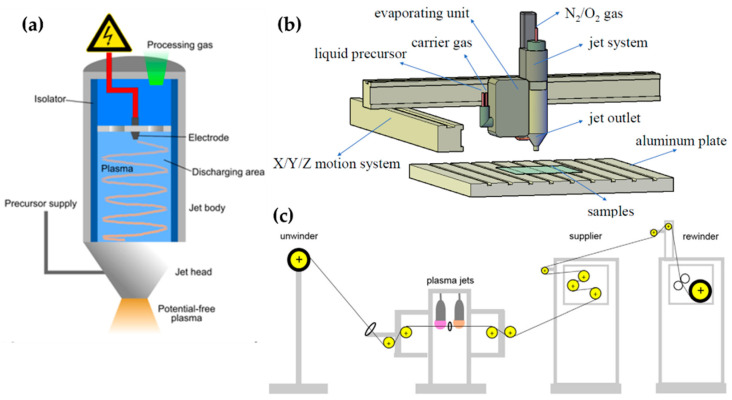
(**a**) The plasma jet instrument schematic (Plasmatreat AS400 with the single-nozzle-type PFW10) [65], and setup of (**b**) polymerization of HMDSO [67] and (**c**) in-line processing of carbon fiber [68].

There are also studies using commercial APPJ devices; Karl et al. [65], Yan et al. [66], and Yang et al. [67] used a commercial plasma jet instrument (Plasmatreat AS400 with the single-nozzle-type PFW10, Figure 5a) to apply a superhydrophobic property to a target substrate by polymerizing hexamethyldisiloxane (HMDSO). A frequency of 19 kHz and a plasma voltage of 285 V were supplied to generate the plasma. The discharge gas (air, N_2_, oxygen (O_2_)) was introduced into this device, while the vapored precursor was transported to the nozzle component by the carrier gas (Ar, N_2_). Yan et al. and Yang et al. put the plasma jet instrument onto a moving system (Figure 5b), and Moosburger-Will et al. demonstrated that this APPJ method is advantageous for in-line processing [68]. They also used the same plasma jet (PFW10) for the deposition of methyltrimethoxysilane onto 1200 m of carbon fiber (Figure 5c). Table 1 presents a summary of this subsection.

### 2.2. Planar Dielectric-Barrier Discharge (DBD) Method

A planar DBD generator is typically a structure in which one or both planar electrodes are covered by dielectric material in order to avoid arc formation while facing one another [69,70]. When an HV current is applied to one side, a glow plasma is created between the electrodes. Since the size of the plasma area depends on the size of the electrodes, it is easier to cover a wider area with this method than with the APPJ method.

**Figure 6:**
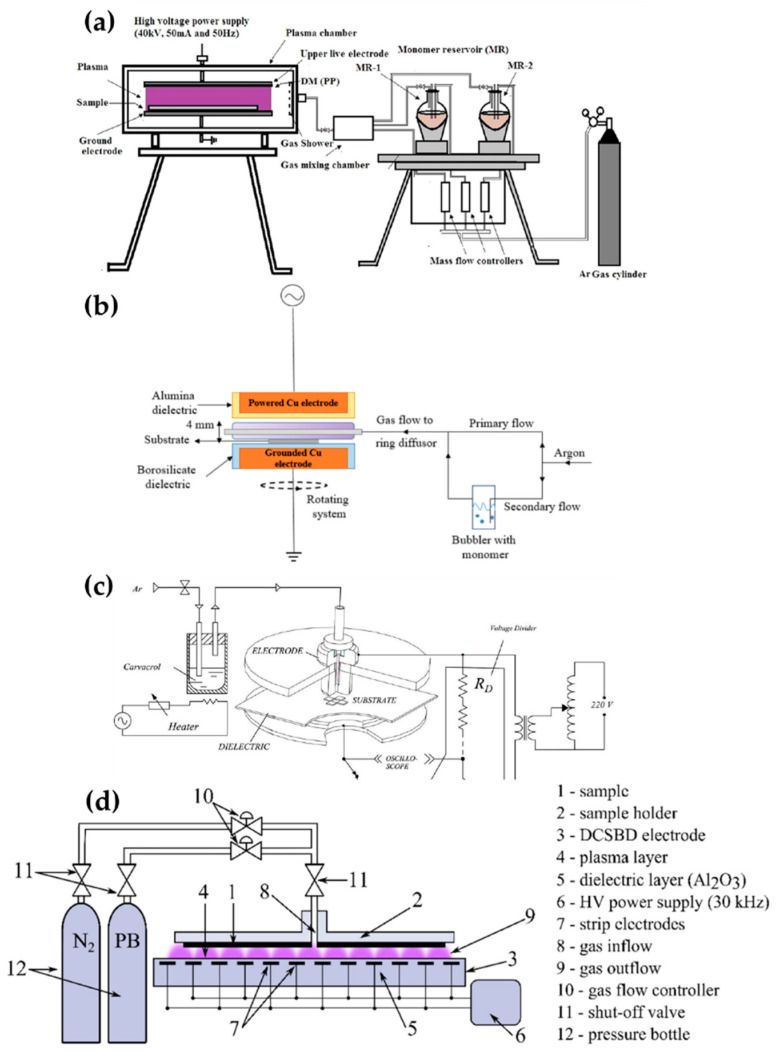
The polymerization systems using planar DBD of (**a**) Pandivaraj et al. [69], (**b**) Mertens et al. [71], (**c**) Getnet et al. [72], and (**d**) Dvorˇáková et al. [73].

Pandivaraj et al. [69], Ramkumar et al. [70], Mertens et al. [71], Getnet et al. [72], and Dvorˇáková et al. [73] report polymer deposition by using planar DBD with fixed electrodes, as shown in Figure 6. Pandivaraj et al. used a plasma generator with a typical DBD structure to increase the antifouling properties of low-density polyethylene (LDPE) films by copolymerizing. This plasma generator consisted of two square electrodes covered by a dielectric material sheet (polypropylene with a thickness of 3 mm) and a chamber. Two electrodes were placed in the chamber with a separation distance of 7 mm. The LDPE film was placed on the lower electrode (grounded electrode), and the plasma was generated by AC power with a voltage of 40 kV and a frequency of 50 Hz through the upper electrode (powered electrode) (Figure 6a). Thereafter, a mixture vapor of acrylic acid and polyethylene glycol (PEG) produced by heat (80 °C and 220 °C respectively) was fed into the chamber [69]. Ramkumar et al. used the same device and procedure as Pandivaraj et al.; however, they employed PEG methyl ether methacrylate (PEGMA) as a precursor to enhance the biocompatibility of LDPE films. The gap between the electrodes was 5 mm and the PEGMA vapor was prepared at 60 °C [70].

Mertens et al. employed two electrodes covered with different dielectric materials for hydrophilic and hydrophobic coatings for about 11 substances. The upper Cu electrode (powered electrode) and the lower Cu electrode (grounded electrode) were covered by a 3-mm-thick α-alumina and a 2-mm-thick borosilicate, respectively. The gap between the two electrodes was 4 mm. This plasma generation system was placed in a Pyrex glass cylinder chamber. The inside of the chamber was pumped down to 270 Pa and filled to atmospheric pressure with Ar. The precursor bubbled by a secondary flow was introduced into the plasma region by diluting it with the primary flow (Figure 6b) [71].

Getnet et al. conducted the deposition of carvacrol thin film on a stainless steel substrate using a DBD generator with only the lower-side electrode (grounded electrode) covered with a polyester sheet as a dielectric layer. Two parallel circular brass electrodes were fixed at 3 mm. A sinusoidal AC pulse with a frequency of 60 Hz and a maximum voltage of 15 kV was applied to the upper electrode (powered electrode). Ar at a flow of 5 L/min was used for the plasma discharge and vaporizing the carvacrol (Figure 6c) [72].

Dvorˇáková et al. used diffuse coplanar surface barrier discharge (DCSBD) methods for fast surface hydrophilization. The DCSBD device was described as a set of parallel, strip-like molybdenum electrodes embedded in alumina as a dielectric material [88]. The thin-layer plasma was generated on the DCSBD surfaces when a high-voltage sine wave was applied to DCSBD electrodes. The substrate holder was fixed at 0.1 mm from the DCSBD surface. A gas mixture of propane, butane, and N_2_ was supplied via a gas inlet in the middle of the substrate holder (Figure 6d) [73].

**Figure 7:**
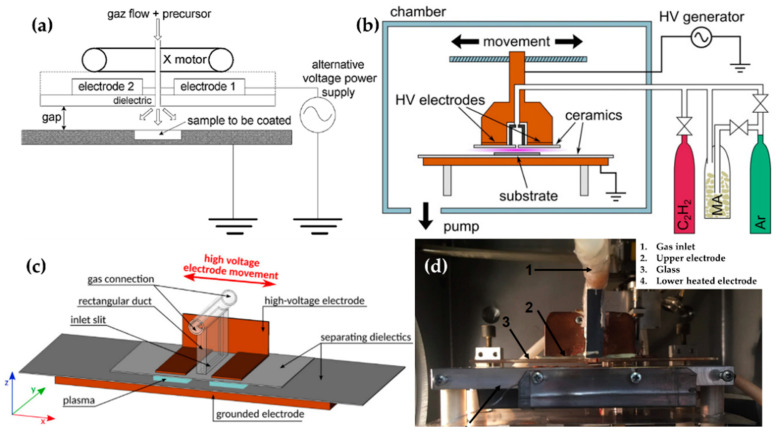
The polymerization systems using planar DBD with a movable top electrode (powered electrode) of (**a**) Bardon et al. [74,89], (**b**) Manakhov et al. [75], and (**c**) Obrusník et al. [76], and the image of the DBD system of (**d**) St’ahel et al. [77].

Bardon et al. [74], Manakhov et al. [75], Obrusník et al. [76], and St’ahel et al. [77] used a movable upper electrode with a gas inlet for improving the deposition uniformity [75], as shown in Figure 7. Bardon et al. improved the coating’s mechanical properties by using DBD plasma and mixtures of dodecyl acrylate (DOCA) with 1,6-hexanediol diacrylate (HdiA) or 1,6-hexanediol dimethacrylate (HdiMA) as precursors. The DBD generator consisted of an earthed-bottom aluminum plate and two powered aluminum top plates covered with a 3.25 mm-thick glass plate. The gap between the bottom electrode and the glass plate was set to 2 mm. The bottom electrode had a slot as a sample holder to set the surface of the samples and the electrode at the same level (Figure 7a) [89]. The precursor mixture was atomized to an aerosol state using He, entering the space between the top electrodes. For the plasma generation, an AC power of 110 W with a peak-to-peak voltage of 11 kV was used [74].

Manakhov et al. utilized a DBD plasma system to copolymerize maleic anhydride (MA) and C_2_H_2_ for carboxyl-rich coatings in a metallic cube chamber. The two rectangular pieces were separated to provide room for the gas feed, while the top electrode was covered with Al_2_O_3_ ceramics with a thickness of 1 mm, and the bottom metallic electrode (grounded electrode) was also covered with the ceramic. The gap between the top and bottom ceramics was set at 1.6 mm (Figure 7b). This chamber was pumped down, and then filled up to 96 kPa with Ar, MA, and C_2_H_2_ as a deposition gas mixture. The plasma was ignited by a sinusoidal wave with a frequency of 5–6.6 kHz and a power of 8 W [75]. Obrusník et al. and St’ahel et al. used a plasma-generating system with the same electrode configuration as Manakhov et al., albeit in open air; the gas was supplied through the inlet in the middle of the top electrode connected to a 4-cm-long rectangular duct (Figure 7c) [76]. In the work of St’ahel et al., a heating spiral was added to the bottom electrode along with a thermocouple to increase the substrate temperature, and the gap between the electrodes was changed to 1.0 mm (Figure 7d) [77].

**Figure 8:**
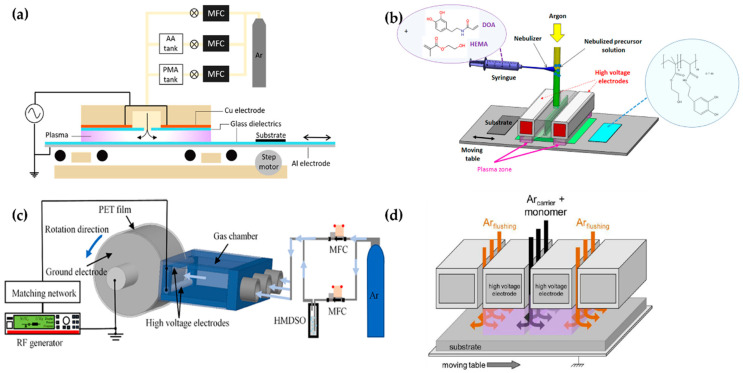
The polymerization systems using planar DBD with a movable substrate stage (bottom electrode) of (**a**) Demaude et al. [78], (**b**) Jalaber et al. [80], (**c**) Ma et al. [81], and (**d**) Loyer et al. [83].

Demaude et al. [78], Nisol et al. [79], Jalaber et al. [80], Ma et al. [81], Ondo et al. [82], and Loyer et al. [83,84,85] employed a DBD plasma generator with a moving substrate stage (bottom electrode) for homogeneous coverage and scale-up [79,81], as shown in Figure 8. Demaude et al. and Nisol et al. synthesized a polymer for hydrophilic/phobic patter coating [78] and age-resistant coating [79], respectively. In this DBD plasma device, a long aluminum strip covered with a 4-mm-thick borosilicate glass plate was used as a movable lower electrode, while the upper electrode consisted of two Cu plates, with a separation between the plates for a gas/precursor inlet. Both the discharge gas and precursor bubbling/carrier gas were Ar (Figure 8a) [78,79].

Jalaber et al. used DBD plasma polymerization for eco-friendly and catalyst-free polymer synthesis. Their device ignited the DBD plasma from between two plane-parallel HV electrodes covered with alumina and a movable substrate stage as a grounded electrode. The distance between the HV electrodes and the substrate stage was 1 mm. A 10 kHz sinusoidal voltage was applied through two plane-parallel electrodes to generate the plasma. Dopamine acrylamide as the precursor was atomized using a nebulizer, and its flow was controlled with a syringe pump (Figure 8b) [80].

Ma et al. utilized a plasma device with a rolling electrode system; two cylindrical HV electrodes (Φ = 4 mm; length = 100 mm) were fixed at a distance of 7 mm from the top layer and the bottom layer of the outlet of the gas chamber. The cylindrical ground electrode (Φ = 60 mm; length = 100 mm) was made of stainless steel and controlled by a motor. Within the triple-inlet gas chamber, some glass wool pieces were placed inside to provide gas flow homogeneity. The ground electrode was wrapped in the PET substrate (Figure 8c). A 13.56 MHz RF power source with 30 W applied through the HV electrodes ignited the plasma in open-air conditions [81].

Ondo et al. and Loyer et al. used a polymerization method called plasma-initiated chemical vapor deposition (PiCVD). This method is characterized by the usage of an ultra-short square-wave pulse power to ignite the DBD plasma for the deposition of a polymer film with a high degree of polymerization [82,83,84,85]. This plasma generator consisted of two parallel HV electrodes made of alumina and a movable stage as the ground electrode [90]. The gap between the parallel electrodes and the ground electrode was maintained at 1 mm (Figure 8d). An ultra-short square-wave pulse was employed to generate the plasma with an extremely low plasma duty cycle (t_on_/(t_on_ + t_off_)); a very low duty cycle plasma (0.1~0.001%) was employed for their studies [82,83,84,85]. Table 2 shows a summary of this subsection.

## 3. Synthesis Method Using Liquid-Type Precursors

APP synthesis methods using liquid-type precursors leverage the interaction between APP and a bulk liquid precursor. The complicated chemical and physical reactions at the plasma–liquid interface cause reduction, oxidation, and sputtering. In most cases, NPs are synthesized from the various radicals generated by the plasma–liquid reactions [43,91]. This method is classified into two types, depending on the location of the APP generation.

### 3.1. Atmospheric-Pressure Plasma (APP) Generated by Outside Bulk Liquid Precursors

APP generated on the outside of bulk liquid precursors—so-called (on-solution plasma)—is affected by natural air components. Therefore, chemical and physical reactions at the plasma–liquid interface take place with various species caused by the interaction between the APP and air components such as O_3_, N_2_O_5_, N_2_O, NHO_3_, H_2_, NO_3_, H_2_O_2_, HNO_2_, and NO_2_ [38]. Complex reactions that are not well understood due to various radicals can produce unusual results [92].

**Figure 9:**
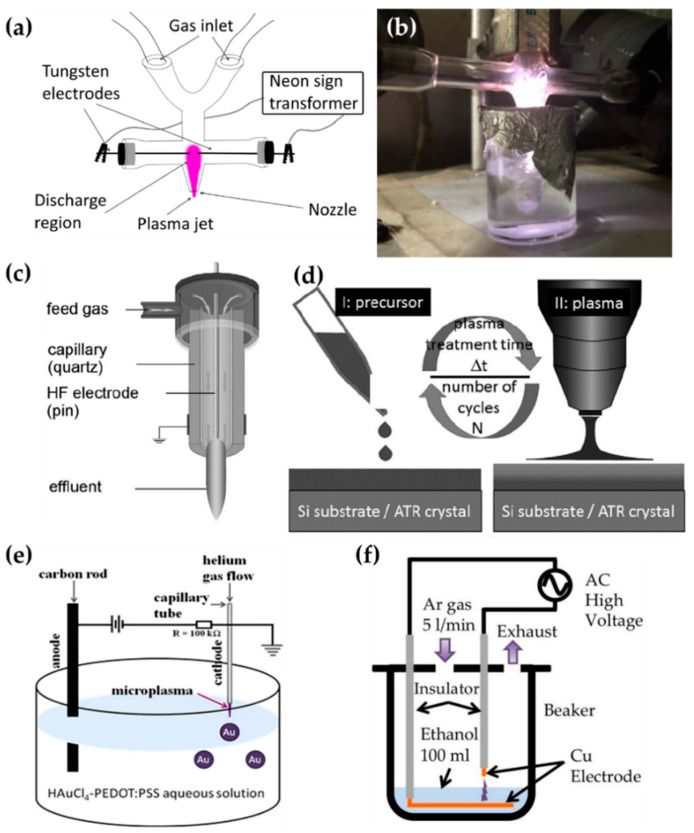
Schematics of generation of the on-solution plasma systems of (**a**,**b**) Tan et al. [93,94], (**c**,**d**) Schäfer [95] et al., (**e**) Zhang et al. [96], and (**f**) Gamaleev et al. [97].

Tan et al. [93,94], Schäfer [95] et al., Zhang et al. [96], and Gamaleev et al. [97] used on-solution plasma generated by various methods, as shown in Figure 9. Tan et al. employed an APPJ with a cross-shaped borosilicate glass body with five nozzles as a plasma outlet, two inlets for Ar flow as a discharge gas, and two side tubes for the electrodes. The power and ground electrodes were made of tungsten, with a separation of 5 mm (Figure 9a). An AC power source with a voltage of 15 kV and a frequency of 60 Hz was applied from a neon sign transformer to ignite the APP. This APP treats the surface of styrene [93] and MMA [94] as bulk liquid precursors (Figure 9b).

Schäfer et al. synthesized three liquid organosilicon compounds (HMDSO, octamethyltetrasiloxane, and tetrakis(trimethylsilyloxy)silane) as precursors using a commercial APPJ (kINPen 11; neoplas tools, Greifswald, Germany). This commercial APPJ consisted of a powered pin electrode centered in a ceramic capillary and a grounded outer ring electrode. Ar gas was introduced as a discharge gas through the ceramic capillary (Figure 9c). A power source of 5 W with a frequency of 1.1 MHz was needed to generate the APP. Liquid precursors were applied to the surface of the substrate, and these were synthesized by exposure to the APP (Figure 9d) [95].

Zhang et al. utilized the discharge between a stainless steel capillary and a liquid bulk surface to synthesize the metallic NPs embedded in a conducting polymer. In this research, the liquid precursor was HAuCl_4_ aqueous solution added to poly(3,4-ethylenedioxy thiophene) polystyrene sulfonate (PEDOT:PSS). This APP generator system used a stainless steel capillary as the powered electrode and a carbon rod as the grounded electrode. The stainless steel capillary also acted on the APPJ generating the He plasma. The distance between the tip of the capillary and the surface of the liquid precursor was 0.9 mm (Figure 9e). The APP was ignited by direct current (DC) with a voltage of 2 kV, and maintained by a voltage of 0.8 kV [96].

Gamaleev et al. employed a pin-type electrode for the generation of on-solution plasma to produce nanographene. First, 100 mL of ethanol liquid precursor was placed in a beaker. A Cu plate grounded electrode was immersed in the ethanol, and a Cu rod powered electrode was placed in the air. The beaker was filled in at a flow rate of 5 slm (Figure 9f) [97]. Table 3 contains a summary of this subsection.

### 3.2. Atmospheric-Pressure Plasma (APP) Generated by Inside Bulk Liquid Precursors

In this section, in-solution plasma is defined as generating the APP in liquid precursors. In-solution plasma is mostly generated between tungsten pin-to-pin electrodes. One of the structurally critical points of the in-solution plasma system is the gap between electrodes; the gap can induce a breakdown of plasma in liquid media [98,99,100]; therefore, tungsten is commonly chosen for synthesis using an in-solution plasma due to its high melting point, corrosion resistance, high stability, and good electrical conductivity [101,102]. Since the in-solution plasma is immersed in the solution, only reactions between the plasma and the solution occur in their entirety. Many recent papers about synthesis methods using in-solution plasma have mostly been reported on the formation of NPs [103,104,105,106,107,108,109,110,111,112].

**Figure 10:**
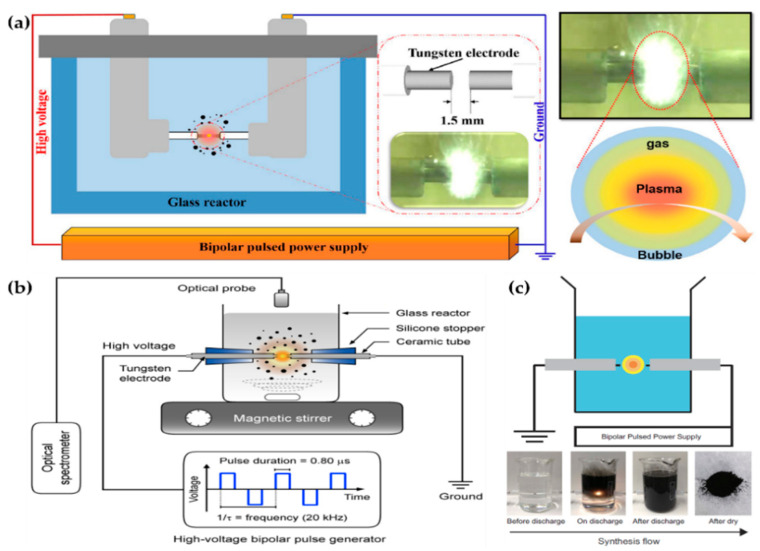
Representative figures on the generation of the in-solution plasma systems of (**a**) Hyun et al. [104], (**b**) Panomsuwan et al. [105], and (**c**) Morishita et al. [106].

**Figure 11:**
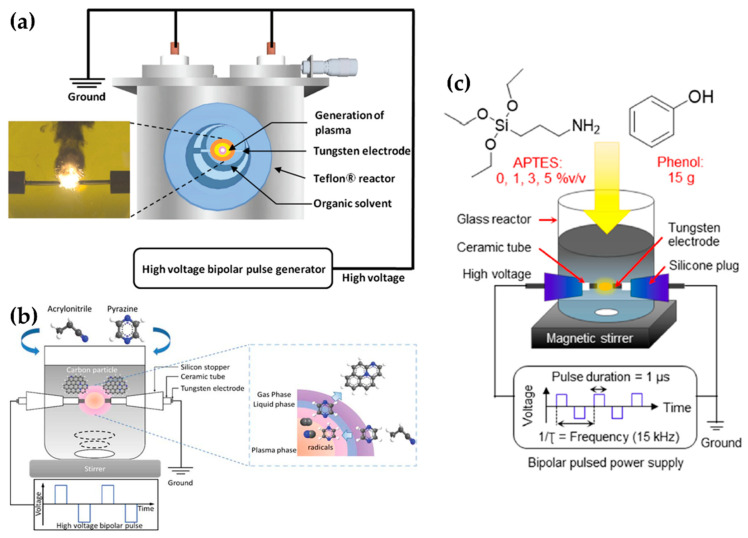
Schematics of generation of the in-solution plasma systems of (**a**) Lee et al. [107], (**b**) Li et al. [108], and (**c**) Tipplook et al. [109].

**Figure 12:**
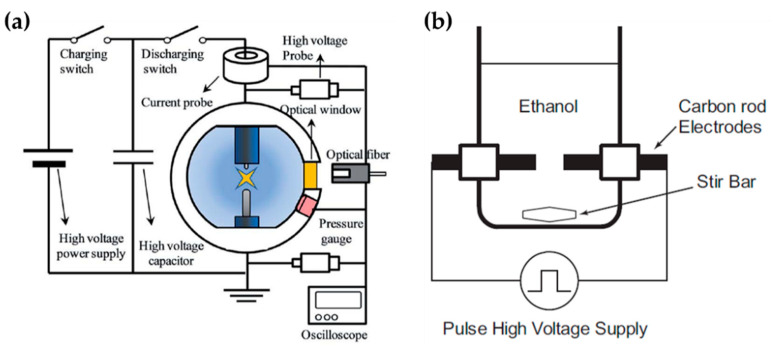
Schematic diagrams of the in-solution plasma systems proposed by (**a**) Lee et al. [91] and (**b**) Li et al. [110].

Hyun et al. [103,104], Panomsuwan et al. [105], Morishita et al. [106], Lee et al. [107], Li et al. [108], Tipplook et al. [109], and Lee et al. [91] used in-solution plasma systems generated by the plasma discharge between tungsten pin-to-pin electrodes, and Alsaeedi et al. [110] chose carbon rods as the electrodes, as shown in Figure 10, Figure 11 and Figure 12. Hyun et al. used a pair of tungsten electrodes 1 mm in diameter to generate the in-solution plasma; these electrodes were located at a distance of 1.5 mm from the glass reactor. A bipolar HV pulse of 2 kV with a repetition frequency of 25–200 kHz and a pulse width of 1 μs was applied via the electrodes. Liquid precursors such as *N*-methyl-2-pyrrolidone, 2-pyrrolidone, pyrrolidine, 1-methylpyrrolidine, pyrrole, cyclopentanone, and cyclohexanone were used for the synthesis of nitrogen–carbon nanosheets (Figure 10a) [103,104].

Panomsuwan et al. also used a pair of tungsten electrodes 1 mm in diameter. The electrodes were covered with an insulating ceramic tube, and had a gap of 1 mm. The bipolar power source used had a pulse duration of 0.80 μs and a frequency of 20 kHz. In-solution plasma was initiated and stably maintained inside 100 mL of liquid precursor under vigorous stirring (Figure 10b). Cyano-aromatic molecules (2-cyanopyridine, cyanopyrazine) were used as liquid precursors for the synthesis of nitrogen-doped carbon NPs (NCNPs) [105].

Morishita et al. also employed tungsten electrodes 1 mm in diameter for in-solution plasma. The electrodes were covered with a ceramic segment, and the distance between them was set to 0.5 mm. The applied voltage was about 1.7 kV, the repetition frequency was 15 kHz, and the pulse width was 1.0 μs. The precursors for the fast formation of nanocarbons were hexane, hexadecane, cyclohexane, and benzene. For the extraction of nanocarbons, plasma-treated precursors were dried at 100 ℃ in an oven (Figure 10c) [106].

Lee et al. selected a pair of tungsten electrodes with a diameter of 0.8 mm, covered with an insulating ceramic tube. The electrodes were placed in the center of the Teflon reactor with a gap distance of 1.0 mm (Figure 11a). The optimized applied voltage conditions of in-solution plasma were found to be 0.5 μs, 100 kHz, and 2 kV for the pulse duration, pulse repetition frequency, and voltage, respectively. In-solution plasma was produced from various solvents—such as carbon, nitrogen, and boron precursors—for synthesizing boron–carbon–nitrogen nanoparticles [107].

Li et al. employed a pair of tungsten electrodes with a diameter of 1 mm as the powered electrode and grounded electrode. These electrodes were placed at the center of a glass reactor with a gap distance of 0.5 mm. To ignite the plasma discharge of the in-solution plasma system, power was applied by using a bipolar pulse with a voltage of 2.0 kV. The pulse duration and repetition frequency were 1 μs and 20 kHz, respectively (Figure 11b). NCNPs were synthesized from pyrazine and acrylonitrile [108].

Tipplook et al. used in-solution plasma as an in situ system for the synthesis of amino-rich nanocarbons. This system had a pair of tungsten rods with a diameter of 1 mm as the powered and grounded electrodes. The electrodes were covered with an insulating tube, inserted into a silicone stopper, and placed at the center of the glass reactor, 1 mm apart. The glass reactor (Figure 11c) contained 100 mL of a liquid precursor (phenol, (3-aminopropyl)triethoxysilane, and ethanol). Then, a HV bipolar pulse power with a voltage of 4kV, a pulse duration of 1 μs, and a repetition frequency of 15 kHz was applied through the electrodes to generate the in-solution plasma [109].

Lee et al. chose an in-solution plasma system with an asymmetrical pair of tungsten needle electrodes. The diameter of the anode was 1.5 mm, and the gap between the electrodes was 5 mm. Five capacitors with a capacitance of 0.1 μF were connected in parallel, and the capacitors with equivalent capacitance of 0.5 μF were charged by a positive-polarity DC power supply with a maximum charging voltage of 19 kV (Figure 12a). Titanium tetraisopropoxide dissolved in ethanol (Ti-contained solution) was used as a liquid precursor to synthesize carbon-incorporated titanium oxide nanocrystals [91].

Alsaeedi et al. reported that nanocarbons were successfully synthesized by using in-solution plasma. In this paper, the in-solution plasma was generated between two carbon electrodes. The carbon rod electrodes each had a diameter of 3 mm, and were separated by a gap of 1 mm. The electrodes were immersed in 50 mL of ethanol (Figure 12b), and the pulse voltage, frequency, and pulse width were 4 kV, 30 kHz, and 4 μs, respectively [110].

**Figure 13:**
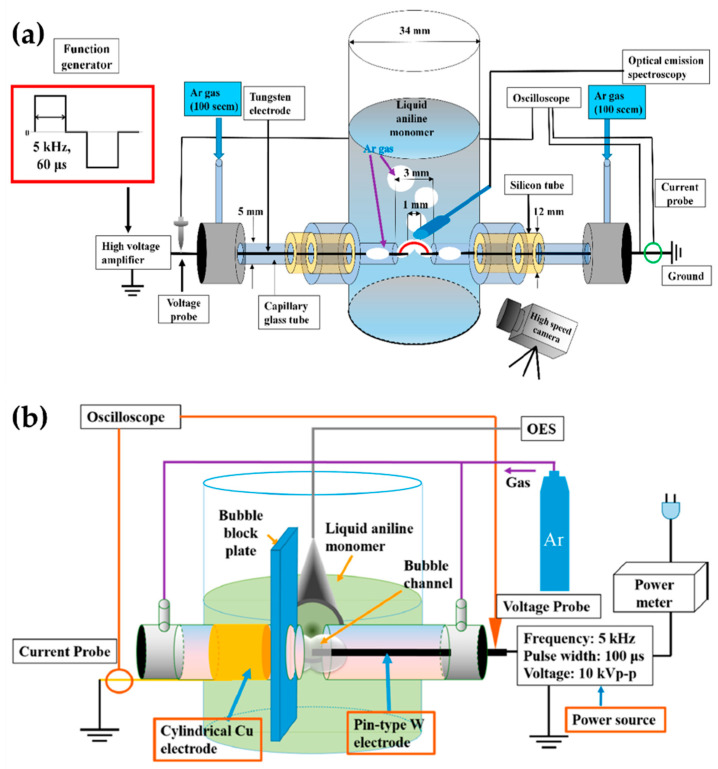
Schematic diagrams of the in-solution plasma system with a gas channel proposed by (**a**,**b**) Shin et al. [111,112].

Shin et al. [111,112] added a gas bubble to the in-solution plasma system. The gas bubble was often used as a plasma channel to enhance in-solution plasma performance in pulsed discharge systems [111]. In a cylindrical glass reactor, the two tungsten electrodes were oriented 1 mm apart, and were positioned in the capillary glass tube for the Ar gas channel. The Ar gas was introduced along with two electrodes in parallel [111], thus forming a gas bubble channel between the electrodes. The gap between the glass capillaries was 3 mm. The plasma discharge was generated in a gas bubble between the electrodes in an aniline monomer acting as the liquid precursor (Figure 13a). A bipolar pulse with an amplitude of 16.4 kV and a frequency of 5 kHz was used to generate the in-solution plasma within the Ar bubble channel, and the bipolar pulse duty ratio was 60 μs [111].

Additionally, Shin et al. [112] also used an asymmetrical electrode structure: the tungsten electrode was placed in a quartz tube for the Ar gas inlet, where its diameter was 0.5 mm and its exact position extruded 1 mm from the end of the capillary quartz tube. On the other hand, a cylindrical copper electrode with a 5 mm width was wrapped around the outside surface of the quartz tube. The copper electrode was positioned 3 mm away from the end of the capillary quartz tube. There were two capillary tubes, with a separation distance of 2 mm. The plasma channel was formed from Ar gas with a flow rate of 100 sccm. A bipolar pulse with a voltage of 16 kV, a frequency of 5 kHz, and a pulse width of 100 μs was employed to generate the in-solution plasma (Figure 13b) [112]. Table 4 displays a summary of this subsection.

## 4. Plasma Polymerization

### 4.1. Synthesis of Polymers Using Plasma Techniques

**Figure 14:**
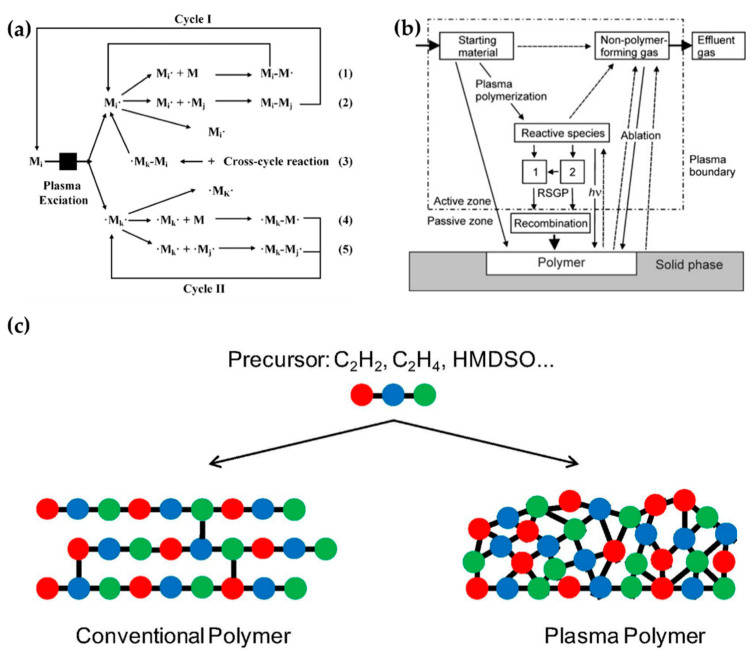
(**a**) Mechanisms of plasma polymerization [113]. (**b**) A scheme of a general plasma polymerization system [114]. (**c**) Comparison of a conventional polymer (left) and plasma polymer (right), derived from equivalent monomers [116].

Plasma polymerization accompanies complex physicochemical reactions that are very different from conventional chemical polymerization methods. In plasma polymerization, monomer molecules are broken into electrons, ions, radicals, and excited molecules through collisions with energetic electrons. A resulting polymer then grows by random recombination among these particles. Figure 14a shows the mechanisms of plasma polymerization [113]; Figure 14b is a scheme of a general plasma polymerization system with these mechanism stages [114]. A polymer is synthesized onto a solid phase through recombination, where single or divalent reactive species generated by plasma are polymerized (recombination) by the reactions between reactive species and monomers (Reactions (1) and (4)) or between the reactive species (Reactions (2), (3), and (5)) [113,114]. Indeed, plasma polymerization is a competitive process between formation by synthesis of polymer-forming species and ablation of the polymer film itself [115]. Unlike conventional chemical polymerization methods—such as self-assembly, layer-by-layer, or spin coating—a continuous fragmentation and random recombination easily creates the high cross-linking property that is characteristic of plasma polymers, as shown in Figure 14c [116]. The high cross-linking property of plasma polymers is responsible for providing better mechanical stability and less morphological changes [74,114,117].

### 4.2. Characterization and Chemical Structure of the Polymerized Films Obtained Using APP Techniques

**Figure 15:**
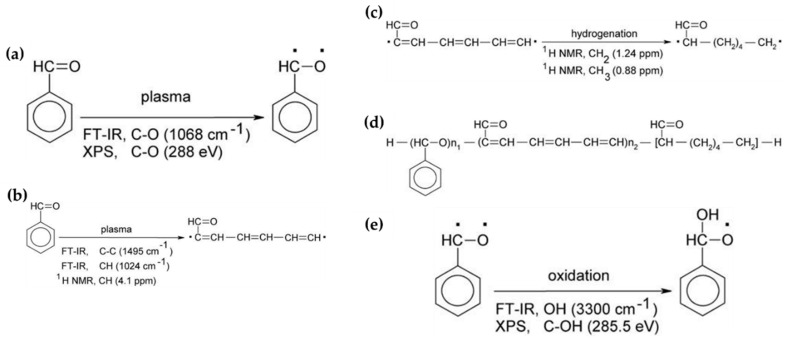
Chemical structures identified via characterization of benzaldehyde-based polymer films using APP techniques, where (**a**) depicts benzaldehyde radical generation by π bond breakage from aldehyde, (**b**) depicts aliphatic chain production by aromatic ring breakage, (**c**) depicts hydrogenation of the aliphatic chain, (**d**) depicts the recombination process between benzaldehyde radicals and aliphatic chains, and (**e**) depicts benzaldehyde radical oxidation under plasma conditions [118].

**Figure 16:**
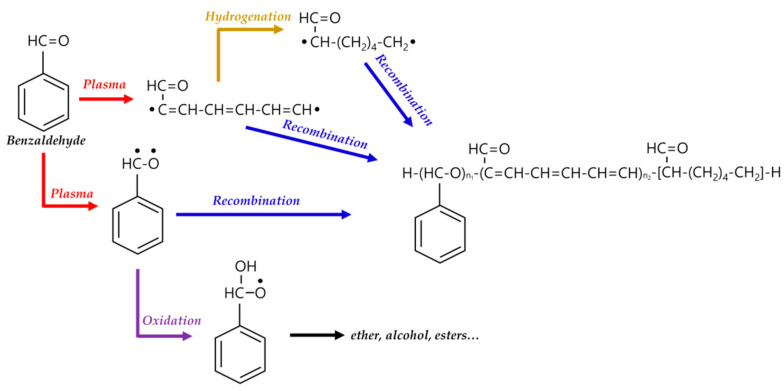
APP polymerization mechanisms, from a chemical point of view, related to the formation of benzaldehyde-based polymer films.

In general, the reaction mechanism of plasma polymerization is mainly established in a vacuum state or low-pressure conditions. In APP methods, the reaction scheme of plasma polymerization is not yet verified, and the related underlying mechanisms are under-studied. Nevertheless, the detailed reaction mechanism of APP polymerization can be confirmed by clearly identifying the chemical structures of polymerized films via characterization methods such as Fourier transform infrared spectroscopy (FT-IR), nuclear magnetic resonance (NMR), and X-ray photoelectron spectroscopy (XPS). The results of this analysis can be seen in Figure 15, where Asandulesa et al. reported a polymerization mechanism by identifying the chemical structures of the polymer films through the characterization of APP-polymerized films synthesized from various monomers [118]. In the case of the benzaldehyde-based polymer films, the polymerized films showed two different types of chemical structures, depending on the plasma conditions: The first type was where the aromatic ring was preserved during the APP polymerization; in this case, reactions of benzaldehyde were initiated by breaking the π bonds from aldehyde, which was confirmed by the presence of C–O bonds in the polymeric film through FT-IR and XPS spectra, as shown in Figure 15a. The second type was where the aromatic ring was broken during the APP polymerization; in this case, aliphatic functional groups were likely obtained from the aromatic ring breakage during plasma polymerization, and aliphatic hydrocarbons (CH_2_ and CH_3_ protons) were also produced by hydrogenation reactions—which was confirmed through FT-IR and NMR spectra, as shown in Figure 15b,c. Finally, the reactive species were shown to randomly recombine, and the polymeric film was obtained via the reactions between radical species during the APP polymerization, as shown in Figure 15d. The reactive species could also be oxidized during film growth, producing functional groups such as ethers, alcohols, and esters—which was confirmed by the presence of ethers, alcohol, esters, and carbonate units in the chemical composition of the polymeric film identified from FT-IR and XPS data, as shown in Figure 15e [118]. In summary, the APP polymerization mechanism, from a chemical point of view, is related to the formation of benzaldehyde-based polymer films, as illustrated in Figure 16.

## 5. Main Properties and Current Applications of the Polymers Obtained via APP Polymerization

**Figure 17:**
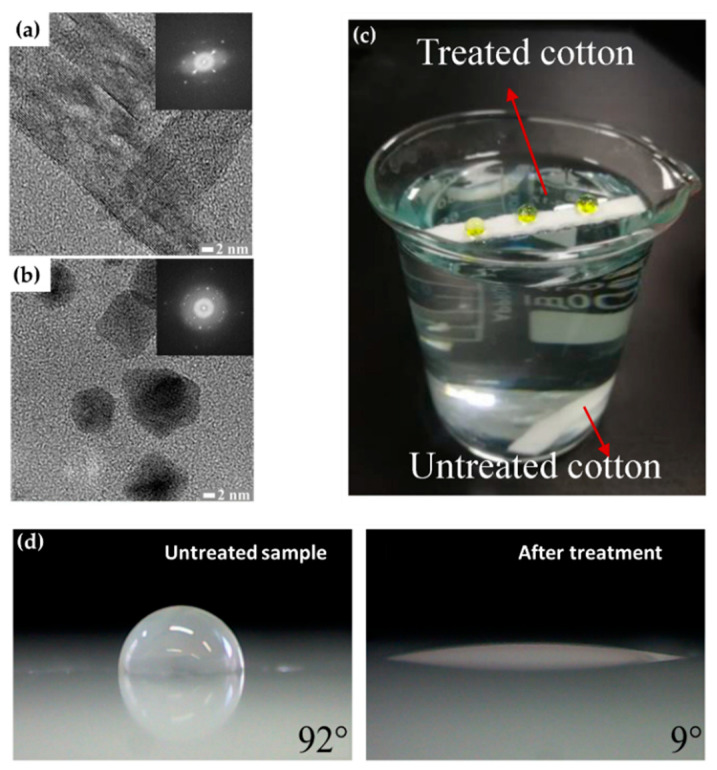
Transmission electron microscopy (TEM) images of polypyrrole NPs with (**a**) single-crystalline and (**b**) polycrystalline properties [62]. (**c**) Superhydrophobicity of the coated films on the cotton fabrics [67]. (**d**) Hydrophilization of the surface of polypropylene [73].

**Figure 18:**
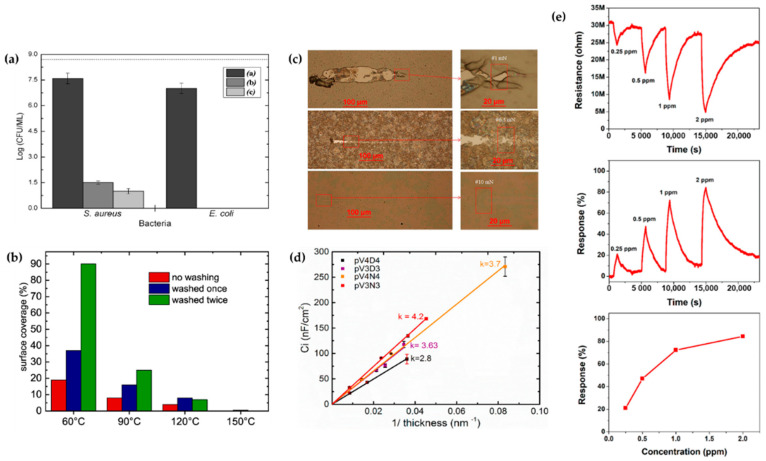
(**a**,**b**) Antibacterial properties created by APP polymerization [72,77]. (**c**) Scratch tracks for coatings of DOCA, HdiA and HdiMA films [74]. (**d**) Estimation of the dielectric constant of insulating layers made by cyclic organosilicons [82]. (**e**) NO_2_-sensing properties of a polythiophene film prepared using the APPJ technique [119].

The technical state of APP polymerization is sufficiently enhanced to synthesize a conducting polymer film with single- and polycrystalline properties (Figure 17a,b) [62]. These advanced APP polymerization techniques are actively investigated for various applications; functional coatings are one such representative application of APP polymerization. Plasma polymerization can change the wettability of any surface to demonstrate hydrophobic/philic characteristics, depending on the amount of polar or nonpolar functional groups on the coating’s surface [78,81]. Therefore, the properties of functional coatings are commonly determined from the kinds of materials used. For example, TMS, APDMES, HMDSO, AA, and PMA are utilized for wettable coatings (hydrophilic/phobic) (Figure 17c,d) [57,67,71,73,78,81]. In a biological application, Getnet et al. [72] and St’ahel et al. [77] investigated whether polymer films synthesized via APP polymerization could be applied for antibacterial properties (Figure 18a,b) [72,77]. As shown in Figure 14c [116], the plasma polymers have the high cross-linking property resulting from continuous fragmentation and random recombination during plasma polymerization; that is, the polymers obtained using plasma techniques show better mechanical stability and fewer morphological changes [74,114,117]. Bardon et al. [74] reported that the DOCA polymer coatings were modified by adding HdiA or HdiMA to the DOCA precursor, and the related mechanical reinforcement was examined (Figure 18c) [74]. Abessolo et al. [82] also reported that the dielectric constant of polymer films deposited by APP could be lowered by varying the monomer precursors used—i.e., siloxane and silazane—as well as by varying their ring size (Figure 18d) [82]. Additionally, the surface morphology of the polymers obtained using APP polymerization shows rough or porous films, thereby enhancing their sensing capability as gas sensors. A porous polythiophene prepared via APP polymerization showed outstanding response properties as a NO_2_ gas sensor when compared to polythiophene synthesized via chemical methods (Figure 18e) [119].

## 6. Conclusions and Future Perspectives

In summary, the plasma synthesis of polymer films and NPs under atmospheric pressure has become an advanced and replicated method, due to the various experimental advantages of this kind of synthesis, such as simplified equipment, faster processes, lower thermal temperatures, lower costs, and eco-friendly waste. In this review, we presented the recent studies on the synthesis of polymer films and NPs using the various APP methods. These methods are categorized into two types, depending on the state of the precursor: The APP methods using a gas/aerosol-phase precursor are almost always used for a polymer deposition or coating. The APP methods using liquid-type precursors are more favorable for the formation of NPs. First, we described and summarized various GATP methods that are employed as synthesis methods for polymer films. These methods all successfully form polymer films via variations in the specific GATP techniques by varying experimental parameters, such as the structure of the electrodes, types of precursors, types of discharge gas, and discharge power sources (frequency, voltage, duty cycle). Next, this review provided a summary and discussion of various liquid-plasma techniques used primarily for the formation of NPs via non-thermal plasma interactions with various liquids, including water or organic monomer solutions. Again, a wide range of NP synthesis can be successfully achieved using liquid-plasma systems with various configurations of electrodes, materials, and plasma power sources.

In the near future, various APP polymer synthesis methods will likely become ideal candidates for industrial applications due to their potential for scaling-up while retaining a practical and sustainable environment for synthesis. However, there remain some challenges for applications of these APP methods, i.e., the low plasma density of APP and the potential for using a large amount of the gas source in some cases. Thus, it is important to incentivize and support further investigations and research on novel APP polymerization and synthesis methods in order to continue to overcome these challenges and accomplish large-scale polymer film deposition or mass production of NPs with stable discharge under open-air conditions.

## Figures and Tables

**Table 1:** Summary of the synthesis of polymers using APPJ methods.

No	Object	Precursor	Power	Year	Author Reference
1	Improvement of flashover performance of polypropylene surface	methyl-methacrylate (MMA)	Ar gas RF power (17 kV, 10~60 kHz)	2020	Zhang et al. [51]
2	Deposition of polymer film from Ar/air/acetylene	Acetylene	Air, Ar gas AC Pulse (Sine) (12 kV, 19 kHz, 2.8 W)	2017	Ricci Castro et al. [52]
3	Deposition of PMMA film	MMA	Ar gas AC Pulse power (23 kHz, 2 W)	2018	Vrekhem et al. [53]
4	Deposition of phosphorous containing functional coatings	Triisopropyl phosphate (TIP)	Ar gas (1 kPa) AC Pulse power (40 kV, 50 kHz)	2019	Pandiyaraj et al. [54]
5	Polymerization of heptylamine	Heptylamine	He gas AC Pulse (Sine) (8 kV, 10 kHz)	2019	Doherty et al. [55]
6	Polymerization of HMDSN	Hexamethyldisilazane (HMDSN)	Air, Ar gas AC Pulse (Sine) (12 kV, 19 kHz, 2.8 W)	2017	Kodaira et al. [56]
7	Superhydrophobic coating	Tetramethylsilane (TMS), 3-aminopropyl(diethoxy)methylsilane (APDMES)	Ar gas RF power (7.5 kV, 11.5 kHz)	2019	Hossain et al. [57]
8	Durable bioactive coating	Laccase	He gas AC Pulse (2∼6 kV, 20 kHz)	2018	Malinowski et al. [58]
9	Copolymerization	Mixture of thiophene and Aniline	Ar gas AC Pulse (Sine) (23 kV, 26 kHz)	2020	Jang et al. [59]
10	Polymerization of PMMA	MMA	Ar gas AC Pulse (Sine) (12 kV, 30 kHz)	2019	Park et al. [60]
11	Humidity-independent conducting polymer	aniline	Ar gas AC Pulse (Sine) (8 kV, 26 kHz)	2017	Park et al. [61]
12	Single-crystalline polymer film	Pyrrole	Ar gas AC Pulse (Sine)(12 kV, 30 kHz)	2017	Kim et al. [62]
13	Conducting polymer film	aniline	Ar gas AC Pulse (Sine) (5 kV, 30 kHz)	2021	Kim et al. [63]
14	Transparent thin film	aniline	Ar gas AC Pulse (Sine) (4 kV, 30 kHz)	2021	Kim et al. [64]
15	Deposition from organosilicon	HMDSO, Tetraethyl orthosilicate (TEOS)	Air, N_2_ gas Pulse power (19 kHz, 1 kW)	2020	Karl et al. [65]
16	Preservation of paper-based relics	HMDSO	Air, Ar gas Pulse power	2019	Yan et al. [66]
17	Superhydrophobic cotton fabrics	HMDSO	O_2_, N_2_ gas RF power (19 kHz)	2018	Yang et al. [67]
18	Reinforcement of carbon fiber	Methyltrimethoxysilane (MTMS)	Ar gas AC Pulse (252 V, 21 kHz, 600 W)	2017	Moosburger-Will et al. [68]

**Table 2:** Summary of the synthesis of polymer films using planar DBD plasma.

No	Object	Precursor	Plasma Source	Year	Author Reference
1	Improvement of antifouling properties	mixture of acrylic acid (AAC) and poly (ethylene glycol) (PEG)	Ar gas AC Pulse (14 kV, 44 kHz)	2019	Pandiyaraj et al. [69]
2	Enhancement of biocompatibility	poly (ethylene glycol) methylether methacrylate (PEGMA)	Ar gas AC Pulse (14 kV, 44 kHz)	2017	Ramkumar et al. [70]
3	Hydrophilic and hydrophobic coatings	11 precursors for hydrophilic and hydrophobic coatings	Ar gas AC Pulse (17.1 kHz)	2020	Mertens et al. [71]
4	Inhibition of bacteria adhesion and proliferation	Carvacrol (5-Isopropyl-2-methylphenol, (CH_3_)_2_CHC_6_H_3_(CH_3_)OH)	Ar gas AC Pulse (15 kV, 60 Hz)	2020	Getnet et al. [72]
5	Surface hydrophilization	propane-butane (PB) gas	N2 gas AC Pulse (30 kHz)	2019	Dvorˇáková et al. [73]
6	Reinforcement of mechanical properties of DOCA film	Dodecyl acrylate (DOCA), 1,6-hexanediol diacrylate (HdiA), 1,6-hexanediol dimethacrylate (HdiMA)	He gas AC Pulse (11 kV, 10 kHz, 110 W)	2018	Bardon et al. [74]
7	Carboxyl-rich coatings	maleic anhydride (MA), acetylene	Ar gas AC Pulse (Sine) (4 kV, 5∼6.6 kHz, 8 W)	2016	Manakhov et al. [75]
8	Copolymerization	MA, acetylene	Ar gas AC Pulse (4 kV, 4 kHz, 3.3 W)	2017	Obrusník et al. [76]
9	Antibacterial properties and cytocompatibility performance	2-methyl-2-oxzoline, polyoxazoline (POx)	Ar gas AC Pulse (6 kHz, 55 W)	2019	St’ahel et al. [77]
10	Hydrophilic/phobic patterns	acrylic acid (AA) propargyl methacrylate (PMA)	Ar, O_2_ gas AC Pulse (Sine) (16.2 kHz, 90 W)	2019	Demaude et al. [78]
11	Age-resistant coatings with tunable wettability	AA, PMA	Ar gas AC Pulse (Sine) (15.64 kHz, 30 to 90 W)	2016	Nisol et al. [79]
12	Preparation of tunable catechol-bearing thin films	dopamine acrylamide (DOA) 2-hydroxyethyl methacrylate monomer (HEMA)	Ar, O_2_ gas AC Pulse (Sine) (10 kHz)	2019	Jalaber et al. [80]
13	Hydrophilicity	HMDSO	Ar gas 13.56 MHz RF (30 W)	2020	Ma et al. [81]
14	Low-k Polymer Insulating Layers	4 cyclic organosilicon monomers	N_2_ gas AC square Pulse (6 kV, 100 Hz)	2019	Ondo et al. [82]
15	Comparison of polymer properties according to applied duty cycle	3 methacrylate monomers (MMA, BMA, GMA)	Ar gasAC square Pulse (6.5 kV, 10 kHz)	2017	Loyer et al. [83]
16	Comparison of polymer properties according to applied duty cycle	2 methacrylate monomers (MMA, GMA)	Ar gas AC square Pulse (6.5 kV, 10 kHz)	2018	Loyer et al. [84]
17	Deposition of NVCL with water-stable and thermo-responsive properties	*N*-vinyl caprolactam (NVCL) ethylene glycol dimethacrylate (EGDMA)	Ar gas AC square Pulse (6.5 kV, 10 kHz)	2019	Loyer et al. [85]

**Table 3:** Summary of syntheses using on-solution plasma methods.

No	Object	Precursor	Plasma Source	Year	Author Reference
1	Plasma-treated styrene	Styrene	Ar gas AC Pulse (15 kV, 60 Hz, 450 W)	2018	Tan et al. [93]
2	Plasma-treated MMA	MMA	Ar gas AC Pulse (15 kV, 60 Hz, 450 W)	2020	Tan et al. [94]
3	Thin, solid SiO_x_ film	HMDSO, octamethyltetrasiloxane (OMCTS), tetrakis(trimethylsilyloxy)silane (TTMS)	Ar gas AC Pulse (1.1 MHz, 5 W)	2017	Schäfer et al. [95]
4	Metallic NPs embedded in a conducting polymer	HAuCl4 aqueous solution, poly(3,4-ethylenedioxy thiophene) polystyrene sulfonate (PEDOT:PSS)	He gas DC Pulse (ignition 2 kV, maintain 0.8 kV)	2017	Zhang et al. [96]
5	Nanographene	Ethanol	AC high voltage	2018	Gamaleev et al. [97]

**Table 4:** Summary of syntheses using in-solution plasma methods.

No	NPs	Electrode (Gap)	Precursor	Plasma Source	Year	Author Reference
1	Nitrogen–carbon nanosheets (NCNS)	Tungsten (1 mm)	*N*-methyl-2-pyrrolidone	Bipolar Pulse (2 kV, 25–200 kHz, 1 μs)	2016	Hyun et al. [103]
2	NCNS	Tungsten (1 mm)	2-Pyrrolidone, 1-methylpyrrolidine, pyrrolidine, pyrrole, cyclopentanone, and cyclohexanone	Bipolar Pulse (2 kV, 200 kHz, 1 μs)	2017	Hyun et al. [104]
3	Nitrogen-doped carbon nanoparticles (NCNPs)	Tungsten (1 mm)	Cyanopyridine, cyanopyrazine	Bipolar Pulse (20 kHz, 0.8 μs)	2016	Panomsuwan et al. [105]
4	Nanocarbons	Tungsten (0.5 mm)	Hexane, hexadecane, cyclohexane, benzene	Bipolar Pulse (1.7 kV, 15 kHz, 1 μs)	2016	Morishita et al. [106]
5	Boron–carbon–nitrogen nanocarbons	Tungsten (1 mm)	Pyridine, B-tribromoborazine, boric acid	Bipolar Pulse (2 kV, 100 kHz, 0.5 μs)	2017	Lee et al. [107]
6	Carbon	Tungsten (0.5 mm)	Pyrazine, acrylonitrile	Bipolar Pulse (2 kV, 20 kHz, 1 μs)	2016	Li et al. [108]
7	Amino-modified nanocarbon	Tungsten (1 mm)	Phenol (3-aminopropyl)triethoxysilane (APTES)	Bipolar Pulse (4 kV, 15 kHz, 1 μs)	2020	Tipplook et al. [109]
8	Carbon TiOX/carbon composite nanosheets	Tungsten (5 mm)	Ti-contained solution	DC (19 kV)	2015	Lee et al. [91]
9	Carbon, Pt–carbon	Carbon (1 mm)	Ethanol	Pulse (4 kV, 30 kHz, 4 μs)	2019	Alsaeedi et al. [110]
10	Polyaniline	Tungsten (1 mm)	Aniline	Bipolar Pulse (16.4 kV, 5 kHz, 60 μs)		Shin et al. [111]
11	Polyaniline	Tungsten–copper (4 mm)	Aniline	Bipolar Pulse (16 kV, 5 kHz, 100 μs)		Shin et al. [112]

## Data Availability

Not applicable.
